# Could It Be Pneumonia? Lung Ultrasound in Children With Low Clinical Suspicion for Pneumonia

**DOI:** 10.1097/pq9.0000000000000326

**Published:** 2020-07-07

**Authors:** Eric Scheier, Nadine Levick, Julia Peled, Uri Balla

**Affiliations:** From the * Department of Pediatrics, Kaplan Medical Center, Rehovot, Israel, affiliated to the Hebrew University, Israel; † Pediatric Emergency Medicine Specialist; ‡ Pediatric Resident; § Director of Research and Innovation in Emergency Health Services, Department of Emergency Medicine, Assuta Medical Center, Ashdod, Israel

## Abstract

**Background:**

Community-acquired pneumonia (CAP) is a significant cause of pediatric morbidity and mortality worldwide. Emergency department point of care ultrasound (POCUS) is a first-line modality for diagnosis of CAP. The current coronavirus disease 2019 pandemic creates a unique opportunity to incorporate lung POCUS into the evaluation of a broader range of children. It has increased the utility of lung POCUS in both evaluation and follow-up of pediatric coronavirus cases. An increased use of lung POCUS creates an opportunity for earlier diagnosis while allowing the opportunity for overdiagnosis of small infiltrates and atelectasis. We collated a case series to demonstrate the benefit of lung POCUS in a very broad range of children.

**Methods:**

We collected a case series of 5 patients between December 2018 and December 2019 who presented nonclassically and were diagnosed with CAP on POCUS by a pediatric emergency physician.

**Conclusion:**

Routine lung POCUS in ill children will allow treating physicians to identify and follow a pulmonary infiltrate consistent with CAP quickly. We anticipate that early and more frequent use of POCUS and earlier diagnosis of CAP may improve outcomes by decreasing healthcare encounters within the same illness and by reducing the incidence of late sequelae of pneumonia such as empyema and effusions. However, we acknowledge that this may come at the expense of the overtreatment of viral infiltrates and atelectasis. Further study is required to improve the specificity of lung POCUS in the evaluation of CAP.

## Abstract 2

Supplemental Digital Content is available in the text.

## INTRODUCTION

Community-acquired pneumonia (CAP) is the cause of 16% of pediatric deaths worldwide, with 10% of cases requiring hospitalization.^1^ Emergency department (ED) point-of0care ultrasound (POCUS) is the first-line modality for diagnosis of CAP.^2^ The current coronavirus disease 2019 (COVID-19) pandemic creates a unique opportunity to incorporate lung POCUS into the physical examination. It has increased the utility of lung POCUS in both evaluation and follow-up of pediatric coronavirus cases.^3,4^ Pediatric coronavirus infections, in contrast with adults, more often present with gastrointestinal symptoms,^5^ and thus COVID-19-associated pneumonia may present atypically.

The Kaplan Medical Center is affiliated with the Hebrew University of Jerusalem and serves a diverse catchment area of over a million people in central Israel. Our pediatric ED (PED) sees just under 30,000 children annually. We use POCUS liberally and present a case series illustrating the utility of POCUS for the evaluation of children with low clinical suspicion for CAP.

## METHODS

**Fig. 1.**
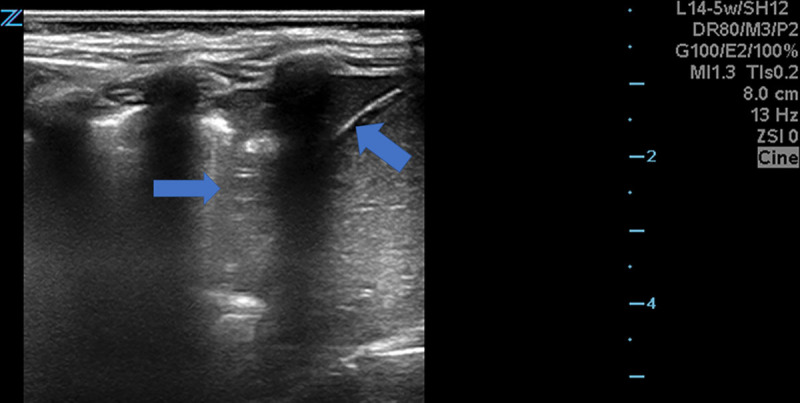
Infiltrate indicated by the rightward arrow. The leftward arrow sits on the spleen with its pointer on the diaphragm. The infiltrate allows visualization of the diaphragm, which is normally obscured by aerated lung. Video Graphic 1. See video, Supplemental Digital Content 1, http://links.lww.com/PQ9/A199. Video Graphic 2. See video, Supplemental Digital Content 2, http://links.lww.com/PQ9/A200. Video Graphic 3. See video, Supplemental Digital Content 3, http://links.lww.com/PQ9/A201.

We collected a case series of 5 patients between December 2018 and December 2019 who presented nonclassically and were diagnosed with CAP on POCUS by a pediatric emergency physician (PEP). Our institutional review board approved the study. A PEP did an ultrasonogram with a Zonare high-frequency probe (ZONARE Medical Systems, Inc., Mahwah, N.J.). We prefer the “lawnmower” approach: a single, continuous scan of the anterior and posterior thorax in a sagittal orientation, including axillae and apices. In all cases, the oxygen saturation and lung examinations were normal, and POCUS identified sonographic consolidations >1 cm with air bronchograms (Fig. 1). Follow-up was done with a search of hospital and outpatient records. The children showed clinical improvement with antibiotics added to the treatment regimen.

### Cases

#### Case 1

A 10-month-old female presented with a single episode of emesis, followed by brief perioral cyanosis. Her mother reported that she has become less active. In the previous week, she had several days of fever and bloody diarrhea and was on day 4 of a 5-day course of azithromycin for dysentery. She had been afebrile since starting azithromycin, and stool had normalized. Her triage temperature was 37.5°C. She was well appearing and had a benign physical examination. POCUS did not show intussusception but a 1.5-cm infiltrate in the left upper lobe, adjacent to the scapula (**see videos 1 and 2, Supplemental Digital Content 1 and 2, *http://links.lww.com/PQ9/A199* and** http://links.lww.com/PQ9/A200). The infiltrate was immediately distal to an irregular, retracted pleura and 2 smaller subpleural infiltrates. A chest radiograph (CXR) was not done. She received amoxicillin.

#### Case 2

A 5-year-old unimmunized male presented with 2 weeks of worsening lower abdominal pain, several days of coughing, recent diarrhea, and an episode of emesis. Upon physical examination, he was afebrile, but he had left lower abdominal pain. A complete blood count (CBC) showed a normal white blood cell (WBC) count with 78% neutrophils. Chemistries and C-reactive protein (CRP) were normal. POCUS of the abdomen was unremarkable. Lung POCUS showed a 1.5-cm infiltrate over the left anterior chest. A CXR confirmed lingular pneumonia. He received azithromycin.

#### Case 3

An 11-year-old male presented with several hours of left shoulder and left abdominal pain. He was afebrile on presentation. He had a single loose stool on the day of presentation, but no emesis. The pain worsened on deep inspiration, and a review of systems (ROS) was significant for cough. He was without fever or tachycardia on presentation, and both lung and abdominal examinations were normal. His pain score was 8/10 by the visual analog scale. POCUS revealed a left-sided infiltrate 5 cm from the pleural line. A CXR confirmed a left upper lobe infiltrate. He received both amoxicillin and azithromycin.

#### Case 4

A 3.5-year-old male presented with fever and back pain. The back pain started before the onset of fever, and a day after a child hit him at daycare. In the evening before presentation, he began to complain of back pain and was febrile to 40°C. A ROS was significant for cough and rhinorrhea. Outpatient CXR performed earlier in the day was read by a radiologist as normal. An outpatient orthopedist felt that he had tenderness over his back and “limited movement” proceeded to the PED for further evaluation. Examination by the resident physician revealed a pain-avoiding gait, but he was without spine or extremity tenderness and had a full range of motion at his back. CBC showed leukocytosis (27,800 cells/µl) with neutrophilia (77%). The erythrocyte sedimentation rate (ESR) was 42 mm/h, and CRP was 20 mg/dl. He was hospitalized with a presumed diagnosis of discitis. On hospital day 2, the consulting orthopedist noted that he had a normal gait and no reproducible tenderness in his back or extremities. Lung POCUS performed at that point found a 2-cm consolidation at the left lung base in the midaxillary line, without effusion (**see video 3, Supplemental Digital Content 3,** http://links.lww.com/PQ9/A201). A CXR was not done. We administered Ceftriaxone while inpatient and discharged after 3 days to complete a course of Augmentin.

#### Case 5

An 11-month-old female born at 30 weeks gestation but without chronic lung disease presented with 2 days of fever to 39.5^o^C, 3 episodes of vomiting, and 4 episodes of watery, nonbloody diarrhea. On examination, she had mild pallor, but the remainder of her examination was unremarkable. WBC was 17,800 cells/µl with a normal differential. A CRP was 2.5 mg/dl. POCUS showed 2 distinct infiltrates in the right upper and right middle lobes to 1.2 cm from the pleura (**see videos 1–3, Supplemental Digital Content 1–3**, http://links.lww.com/PQ9/A199, http://links.lww.com/PQ9/A200***, and*** http://links.lww.com/PQ9/A201). CXR, as read by our pulmonologist, revealed a subtle infiltrate of the right upper lobe. We administered 3 days of ceftriaxone and IV fluid therapy in the hospital, followed by amoxicillin’s outpatient course. Inpatient examination was notable for prolonged expiratory phase, diffuse rhonchi, and tachypnea. We detected adenovirus in stool and nasopharyngeal wash.

## DISCUSSION

CAP is a significant cause of pediatric morbidity and mortality worldwide.^1^ The diagnosis of CAP is based on clinical criteria.^6,7^ CXR is a poor gold standard for CAP due to low sensitivity, fair interrater reliability, and an inability to distinguish viral from bacterial infiltrates.^8^ However, the CXR is useful in context. A recent study of children presenting to the PED with CAP showed that hypoxemia, physical examination findings, and chest imaging findings predicted the need for medical interventions while laboratory assessment did not.^9^ It stands to reason that POCUS in this context could replace CXR, and that an assessment of CAP severity can 1 day be made in the outpatient setting. A recent review of over 6 million outpatient diagnoses of CAP from 2008 to 2015 showed that radiography was used in only 43% of cases.^10^ In the emergency department, CXR use remains high—despite a mild decrease over the prior decade, 80.4% of CAP diagnoses in 2018 involved CXR.^11^

Portable lung POCUS in the PED allows for the evaluation of lungs in real-time without ionizing radiation.^12^ There is a theoretical risk of pulmonary capillary hemorrhage when the mechanical index is above 0.4 (our lung preset creates a mechanical index of 1.3). However, the clinical significance of this is unclear.^13^ POCUS has better sensitivity with similar specificity when compared to CXR for pediatric CAP.^1,14,15^ Specificity for CAP is high when consolidations exceed 1 cm.^16,17^ Lung POCUS by PEP is associated with decreased PED length of stay and cost compared with CXR,^18^ and can be used repeatedly to monitor the progression of an infiltrate.^19^ However, like CXR, pediatric lung POCUS has only moderate interrater reliability.^20^

Although a physician experienced in POCUS evaluated the children in this series, novice practitioners after focused training can use this tool accurately, with operator experience improving accuracy.^21,22^ Further development and market penetration of high-quality handheld ultrasound devices and artificial intelligence tools such as those studied in the interpretation of CXR^23^ will allow for junior physicians to routinely perform POCUS evaluations as an extension of the physical examination. Although we are not suggesting that all children require POCUS screening for CAP, we believe that the increasing availability of this tool allows for hybrid physical examinations that combine auscultation with insonation in children with low pretest probability for CAP.

Although our POCUS examinations were brief, the time required to perform the scan adds additional time to the treating physician’s evaluation.^2^ Consolidations not extending to the pleura or in areas covered by bony structures may be difficult to view. There is a potential for overdiagnosis of CAP due to ultrasonography’s ability to detect small consolidations of uncertain relevance. Even larger consolidations may be viral, and we must rely on clinical context to determine whether they require antibiotic treatment. In the study of Lissaman et al,^17^ 23% of patients with sonographic consolidation improved without antibiotics. In a larger study, 74% of children with outpatient CAP diagnoses received antibiotics, indicating that 26% of children with CAP were discharged without antibiotics.^10^ In a separate study, repeat PED visits were no higher for children with CAP discharged with antibiotics than without.^24^ Thus, CXR is an imperfect predictor of CAP requiring treatment.

Additionally, distinguishing infiltrate from atelectasis on ultrasound requires the identification of dynamic rather than static air bronchograms, which can be challenging on a small, moving child. There continues to be a dearth of literature on this point—the initial distinction by Lichtenstein et al^25^ of consolidation from atelectasis on sonography was made on adult intubated patients. Lichtenstein et al^25^ recently created the BLUE protocol in adults to distinguish between consolidations arising from hemodynamic pulmonary edema, pulmonary embolism, atelectasis, contusion, tumor, drowning, and pneumonia.^26^ No such classification exists for children. A study of 40 children with neuromuscular disease showed an excellent agreement between atelectasis on POCUS by either a radiologist or pediatrician with experience in this modality and CXR as interpreted by a radiologist, with the qualification that clinical correlation is required in the not infrequent case of an inconclusive result.^27^ A study of 80 intubated neonates in intensive care, using expanded criteria for the sonographic diagnosis of atelectasis, showed a higher sensitivity for atelectasis on lung ultrasound than on CXR, using CT as a gold standard when CXR was normal.^28^ Last, a study of 15 pediatric patients anesthetized for MRI showed 88% accuracy when comparing lung ultrasound for atelectasis with MRI, with high interrater reliability for both modalities.^29^ Further study is required to establish the accuracy of lung POCUS in differentiating atelectasis from pneumonia on a broader scale in children, especially if the pretest probability for pneumonia is low. When in doubt, we relied on the presence of surrounding pleural irregularities and B-lines to establish that the infiltrate is pneumonia. In the PED, without continuity of care, the POCUS practitioner should err on the side of caution and show convincingly that the infiltrate is viral or is, in fact, atelectasis rather than discharge the patient with untreated CAP.

This case series illustrates that neither laboratory results nor symptoms such as lower abdominal pain, back pain, or gastrointestinal symptoms exclude CAP. The majority of our cases were afebrile, some with a single episode of emesis or several hours of discomfort. These children would likely have had repeat health care encounters, more laboratory examinations, radiography, and delayed diagnoses without the use of POCUS. With COVID-19 presenting with a range of symptoms, availability of handheld ultrasound facilitates repeat lung examinations, often by a single practitioner, without incurring discomfort or radiation exposure.

Lung POCUS can and should be done by a practitioner in an inpatient or outpatient setting with access to the technology and training deemed adequate for POCUS credentialing by their facility. We can examine children of all ages using a low-frequency curvilinear probe. However, a high-frequency linear probe, with limited depth but higher resolution, may be more helpful in younger children. POCUS can replace radiographs, especially in the early days of illness, when CXR may still be normal. In the outpatient setting, infiltrates can be followed during treatment to show resolution or, in the case of continued fever, to assess for complications without exposure to ionizing radiation.

Ultrasound of the lungs is not a new modality. Indeed, the ultrasound probe was described in the radiology literature over 30 years ago as “the next stethoscope.”^30^ With time, ultrasound units became smaller and more portable, with many current models small enough to be pocketed. However, CXR remains the primary imaging modality for CAP in both the outpatient and PED settings, and descriptions of lung POCUS use in the literature are generally reserved for patients with more classic presentions of CAP such as fever, cough, or abdominal pain. As ultrasound machines become smaller and less expensive, we may see increased use in these settings. Some have suggested that handheld lung ultrasound may be used as an adjunct to the physical examination,^31^ which may lead to infiltrates of all sizes discovered earlier in the course of illness and in a wider spectrum of presentations, particularly as our understanding of the varied presentations of COVID-19 in children evolves. Further study is required to establish that the novice practitioner can use POCUS to scan the lung with test parameters comparable to radiography to identify the POCUS characteristics of atelectasis in children and CAP in subcentimeter infiltrates, and to find the right balance between overtreatment of benign sonographic findings and early treatment of CAP.

## CONCLUSIONS

Lung POCUS is now a well-established diagnostic tool for children presenting to the PED with signs and symptoms concerning for CAP. The current COVID-19 pandemic creates an opportunity to improve the availability of lung POCUS throughout the hospital. Routine lung POCUS in ill children will allow treating physicians to identify and follow a pulmonary infiltrate consistent with CAP quickly. We anticipate that early and more frequent use of POCUS and earlier diagnosis of CAP may improve outcomes by decreasing healthcare encounters within the same illness and by reducing the incidence of late sequelae of pneumonia such as empyema and effusions. However, we acknowledge that this may come at the expense of the overtreatment of viral infiltrates and atelectasis. Further study is required to improve the specificity of lung POCUS in the evaluation of CAP.

## DISCLOSURE

The authors have no financial interest to declare in relation to the content of this article.

## Supplementary Material


